# Deciphering internal and external factors influencing intestinal junctional complexes

**DOI:** 10.1080/19490976.2024.2389320

**Published:** 2024-08-16

**Authors:** Zachary Markovich, Adriana Abreu, Yi Sheng, Sung Min Han, Rui Xiao

**Affiliations:** a Department of Physiology and Aging, College of Medicine, University of Florida, Gainesville, FL, USA; b Graduate Program in Biomedical Sciences, College of Medicine, University of Florida, Gainesville, FL, USA; c Center for Smell and Taste, University of Florida, Gainesville, FL, USA; d Institute on Aging, University of Florida, Gainesville, FL, USA; e Genetics Institute, University of Florida, Gainesville, FL, USA; f UF Health Cancer Center, University of Florida, Gainesville, FL, USA

**Keywords:** Gut barrier, junctional complex, microbes, intrinsic modulators, extrinsic modulators, C. elegans, Drosophila, mouse model, Gastrointestinal diseases

## Abstract

The intestinal barrier, an indispensable guardian of gastrointestinal health, mediates the intricate exchange between internal and external environments. Anchored by evolutionarily conserved junctional complexes, this barrier meticulously regulates paracellular permeability in essentially all living organisms. Disruptions in intestinal junctional complexes, prevalent in inflammatory bowel diseases and irritable bowel syndrome, compromise barrier integrity and often lead to the notorious “leaky gut” syndrome. Critical to the maintenance of the intestinal barrier is a finely orchestrated network of intrinsic and extrinsic factors that modulate the expression, composition, and functionality of junctional complexes. This review navigates through the composition of key junctional complex components and the common methods used to assess intestinal permeability. It also explores the critical intracellular signaling pathways that modulate these junctional components. Lastly, we delve into the complex dynamics between the junctional complexes, microbial communities, and environmental chemicals in shaping the intestinal barrier function. Comprehending this intricate interplay holds paramount importance in unraveling the pathophysiology of gastrointestinal disorders. Furthermore, it lays the foundation for the development of precise therapeutic interventions targeting barrier dysfunction.

## 1. Introduction

The digestive system is the first organ to develop during animal evolution.^1^ While many species can thrive without specialized organs like the brain, heart, kidney, or lung, none can endure without a functioning gut. In fact, the digestive system emerges as the linchpin of survival across multicellular organisms.^1^ In humans, the gastrointestinal (GI) tract stands as the largest interface between the organism and its environment, spanning an estimated area of 20–30 square meters.^2^ This vast expanse of the adult human intestinal epithelium forms a critical barrier, akin to the protective function of the skin albeit on a much larger scale.^2,3^ Within this intricate ecosystem, an estimated 100 trillion to over 1,000 trillion microbes reside, many in symbiosis with the host by supporting metabolism and competing against pathogens. Despite this symbiosis, numerous microbes also constantly pose a threat of pathogenic invasion, which can severely disrupt bodily balance. To maintain stability amidst this constant assault, the intestine must uphold a strong barrier, tightly regulating the entry of foreign particles into the body.^4,5^

Arguably the most important role of the intestinal epithelium cells (IECs) is their highly-tuned selectivity for the passage of molecules across the intestinal epithelium, a single-cell thick layer of tissue that is selectively permeable to different molecules depending on their size and charge.^6,7^ The ‘Leak Pathway’ describes a route in which larger, noncharged molecules (>5 Å) can move across the barrier (albeit in limited capacity), whereas the “Pore Pathway” allows for smaller (~4.5 Å), charged ions and water molecules to permeate. ^8–10^ While these pathways differ in their functions, they are controlled by a large group of evolutionarily conserved protein structures with similar features that form connections to anchor adjacent cells, namely, intestinal junctional complexes.^7^

Disruptions in the integrity of the intestinal barrier frequently result in the abnormal leakage of gut contents into the bloodstream, triggering increased inflammation, autoimmune reactions, and malnutrition, among other effects.^11,12^ This phenomenon is implicated in a broad spectrum of diseases, including inflammatory bowel diseases (IBDs), irritable bowel syndrome (IBS), liver disorders, and even neurodegenerative conditions. ^13–18^ The increasing evidence linking intestinal permeability with IBDs, alongside their significant economic burden, has prompted researchers to propose targeting the intestinal barrier as a strategy to manage various intestinal and extraintestinal ailments. Notably, studies indicate that increased intestinal permeability often precedes the onset of IBDs and other related conditions, suggesting a potential causal role.^13,19^ For instance, longitudinal studies with first-degree relatives of Crohn’s Disease patients show that these individuals often experience increased permeability years before diagnosis.^19^ Additionally, research suggests a positive correlation between the severity of IBD cases and elevated permeability levels.^13^

IBDs are prevalent in developed nations, affecting over 0.3% of the population, with an estimated 3 million adults diagnosed in the United States alone by 2015.^20,21^ In 2004, the direct medical costs for IBD patients in the United States surpassed $6 billion annually, while Europeans incurred direct costs ranging from €4.6–5.6 billion despite a lower incidence rate.^22,23^ Given the significant implications of intestinal permeability in IBDs and related conditions, understanding how junctional complexes regulate intestinal permeability is of utmost importance. In this review, we focus on the intestinal junctional complexes by firstly examining their components across different species, and then exploring techniques for assessing intestinal permeability. Subsequently, we survey some of the known signaling pathways and microbial elements involved in influencing intestinal permeability and maintaining gut barrier integrity. Lastly, we spotlight the intricate relationship between the gut microbiome, particularly bacteria, in both fostering and protecting against leaky gut conditions, highlighting recent advances and posing questions for the future of the field.

## 2. The structure and organization of the intestinal barrier complexes

### 2.1. Mammalian intestinal barrier complexes

**Figure 1.**
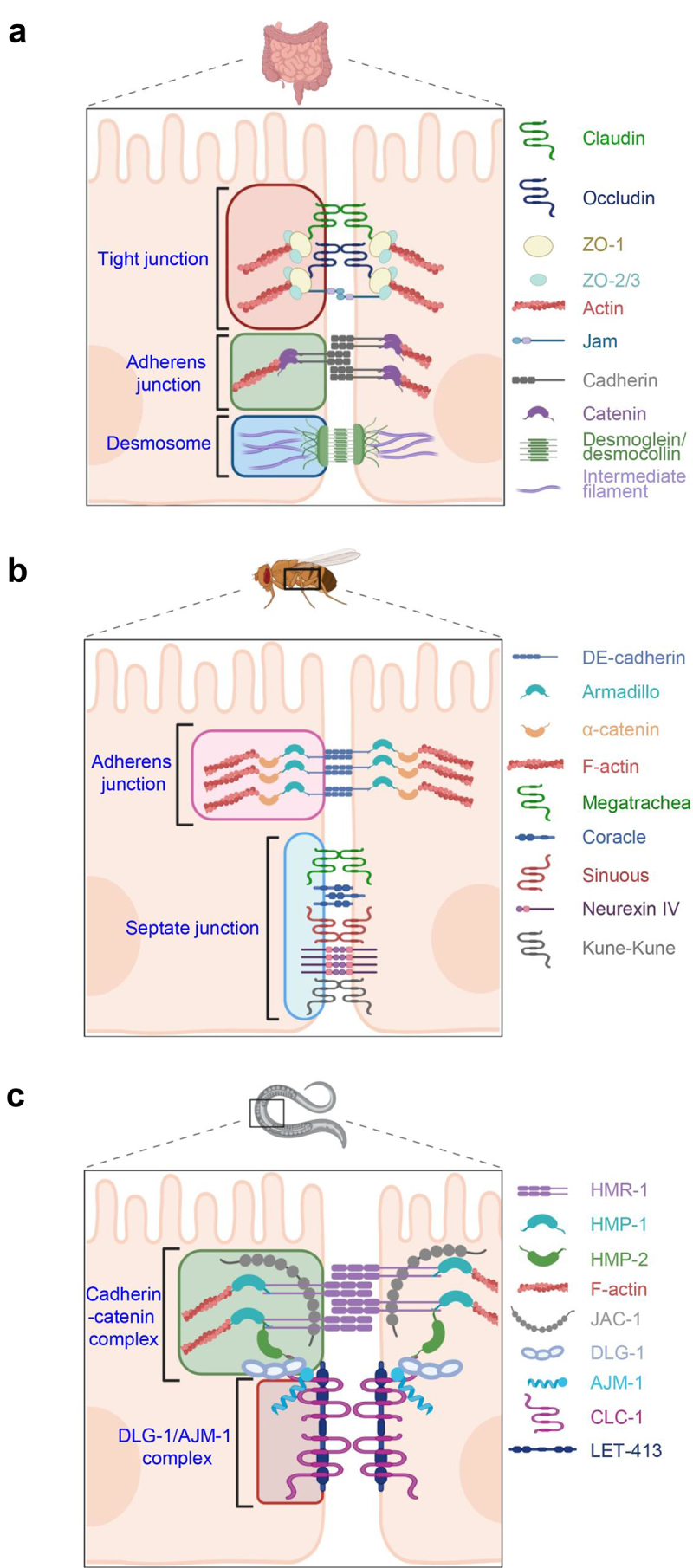
Comparative analysis of junctional complexes across evolutionary models of intestinal barriers. (a), in the vertebrate intestine, junctional complexes comprise tight junction, adherens junction, and desmosomes, with each complex containing multiple key junctional proteins. (b), in the *Drosophila* intestine, adherens junctions and septate junctions form the protective junctional complex among intestinal epithelial cells. Many evolutionarily conserved junctional proteins are expressed in the fly intestinal barrier. (c), the *C. elegans* apical junction consists of the cadherin-catenin complex and DLG-1/AJM-1 complex, with many junctional proteins sharing homology with higher species.

The mammalian intestinal barrier, traditionally conceptualized as a tripartite junctional complex, comprises tight junctions, adherens junctions, and desmosomes (Figure 1(a)).^6,24^ In mammalian cells, the most apical connection between intestinal cells is the tight junction, a protein complex that forms a firm seal between neighboring cells.^25^ Also known as *zonulae occludens*, tight junctions were first described in the mid-1960s.^24^ A characteristic of these junctions is the fusion of adjacent cell membranes at multiple points along the cells’ surface. At these points of fusion, the intercellular space is eliminated, forming a very tight seal between cells.^24,26^ In addition, the tight junction acts as a “fence” on the cell membrane that separates the apical and basolateral sides and controls the traffic of components across the cell membrane.^7,27^ Their formation is dependent on the expression of several proteins that vary in size and function. Foremost are the zonula occludens proteins (ZO-1, ZO-2, and ZO-3), a family of scaffolding proteins that anchor other transmembrane proteins to the cytoskeleton. ZO-1, the primary member of this family, is hypothesized to play a crucial role in regulating tight junction structure. It functions as a bridge linking transmembrane proteins like claudin and occludin to the actin cytoskeleton.^28,29^

Claudin proteins are integral membrane proteins that span the cell membrane four times and extend outward to engage in either homophilic or heterophilic interactions with other claudins.^30^ The mammalian claudin family consists of approximately 27 different proteins with varying functions. While the functions of many claudin family members remain unknown, several (such as claudins 1, 3, 4, 5, 7, 11, 14, and 19) are known for their sealing properties, which enhance the integrity of tight junction barriers.^31–33^ By contrast, other claudins are not involved in tight junction sealing, rather forming selective channels that allow for the passage of cations (claudins 2, 10b, 12, and 15), anions (claudins 10a and 17), and water molecules (claudin-2) across tight junctions.^30,31,34^ Nonetheless, all claudin proteins play crucial roles in the formation of tight junctions, and ongoing research aims to uncover the functions of the remaining family members.^30,31,34^

Occludin is a member of the tight junction-associated MARVEL-domain protein (TAMP) family of proteins. Despite differences in length and the specific cytosolic domains of the proteins, occludin and other TAMPs function similarly to claudins in their membrane transversal and pairing with other proteins in the tight junction.^30^ The pairing of these proteins along with other tight junction proteins such as Junctional Adhesion Molecules (JAMs, which span the membrane only once) and tricellulin leads to the formation of a barrier that prevents the free flow of molecules across epithelial membranes.^30^

More basal than tight junctions are the adherens junctions, also known as *zonulae adherens* (Figure 1a). Unlike tight junctions, adherens junctions do not fuse the membranes of adjacent cells and instead leave a gap between the cells approximately 20 nm in length.^35^ Protruding into the gap and bridging the cells are 9 nm thick, rod-shaped proteins known as cadherins.^35^ Cadherin proteins are anchored into the membrane and form binding pairs with other cadherins on neighboring cells.^35,36^ Within the cell they bind catenin proteins through which they are associated with the actin cytoskeleton, ultimately providing a support network and rigidity for the cellular junction.^36^ While their morphology differs in different cell types, adherens junctions in polarized epithelial cells, like those found in the intestine, form a continuous ring with intracellular F-actin (known as the adhesion belt) that wraps around the entirety of the cell and contributes to the barrier function of the tissue.^36^

Closest to the basal surface of the cell and the last of these three junctional complexes are desmosomes (Figure 1(a)), also recognized as *Macula Adherens*.^37^ In contrast to adherens junctions reliance on actin microfilaments, desmosomes utilize intermediate filaments.^38^ Bundles of intermediate filaments extend outwards from the nucleus to the cell membrane where they anchor the desmosome junctional proteins.^38^ Nonetheless, desmosomes are similar to adherens junctions in that desmosomes also employ a family of cadherin proteins to mediate their junctional capacity. Within the intercellular space, two cadherin subtypes (desmogleins and desmocollins), recognize and bind to each other, fostering a robust cell-to-cell junction that withstands mechanical stress.^38,39^

### 2.2. Invertebrate intestinal barrier complexes

Many of the intestinal junctional proteins found in mammals are evolutionarily conserved across diverse species, including lower organisms. For instance, the model organism *Drosophila melanogaster* (*D. melanogaster*) possesses analogues of both adherens junctions and tight junctions, although their organization differs from vertebrates. In *D. melanogaster*, the adherens junction occupies the most apical position among junctional complexes (Figure 1(b)).^40^ It is predominantly composed of *Drosophila* epithelial (DE)-cadherin, which serves as a counterpart to vertebrate cadherin proteins.^40^ DE-cadherin circumscribes the cells near the apical surface and interacts with the sole *D. melanogaster* β-catenin homologue, known as *Armadillo*.^41^ Similar to mammalian adherens junctions, the binding of DE-cadherin to Armadillo β-catenin facilitates interaction with α-catenin, which in turn directly interacts with actin cytoskeleton.^41^ In contrast to mammalian tight junctions, the analogous structure in *D. melanogaster* is located subapical to the adherens junction, known as the “septate junction” (Figure 1(b)).^42^ Septate junctions serve a similar function to tight junctions by sealing cells together to form a barrier across epithelia.^43^ Similar to tight junctions, septate junctions contain claudin proteins such as Megatrachea (Mega), Sinuous (Sinu), and Kune-Kune that regulate the structure and function of the junction.^42^ They also contain two important structural proteins named Neurexin IV and Coracle.^43^ While mammalian tight junctions appear as fused regions between adjacent cells, septate junctions have a ladder-like intercellular structure and are not as tightly associated.^42^ Overall, while the organization of junctional complexes in *D. melanogaster* differs from that in mammals, many homologous proteins function similarly to create an protective cell barrier.

In *Caenorhabditis elegans* (*C. elegans)*, the intestine consists of just 20 cells that together form a tubular structure within the organism.^44^ Despite their apparent simplicity, these intestinal cells are interconnected via an apical junctional complex of remarkable sophistication. As a functional homologue to the bipartite cell junctions in the *Drosophila* intestine (adherens junctions and septate junctions) and tripartite structures in vertebrate intestines (tight junctions, adherens junctions, and desmosomes), the *C. elegans* Apical Junction (CeAJ) exhibits a more condensed structure, featuring two distinct domains: the cadherin-catenin complex (CCC) and the DLG-1/AJM-1 complex (DAC) (Figure 1(c)).^44^ The CCC in *C. elegans*, akin to classical adherens junctions in vertebrates, comprises proteins such as HMP-1, HMR-1, and HMP-2 which are homologues of human α-catenin, E-cadherin, and β-catenin, respectively.^45^ Notably, HMP-1 directly binds to F-actin and associates with HMR-1 via HMP-2, mirroring the interactions observed in mammalian cells.^45^ Additionally, the Juxtamembrane Domain-associated Catenin (JAC-1), a homologue of human p120 catenin, plays a crucial role in regulating cadherin function, as evidenced by exacerbating morphological defects in HMP mutants when absent.^46^ More basal in the CeAJ is the DAC which is formed by the two key proteins: AJM-1 and DLG-1. AJM-1, an orthologue of human AJM1, is a coiled-coil protein, while DLG-1 shares homology with the *Drosophila* discs-large protein.^47^ DLG-1 aids in recruiting AJM-1 to the DAC, with its spatial localization mediated by another protein, LET-413.^47^ Mutations in LET-413 result in severe adhesion and polarity defects, emphasizing its role in proper protein positioning.^48^ The DAC also contains a homolog of mammalian claudin named CLC-1.^44^ Together, the CCC and DAC establish a densely packed region between *C. elegans* intestinal cells, mirroring the junctional complexes observed in mammalian intestines and featuring numerous homologous proteins.

### 2.3. Animal models for studying intestinal permeability and related conditions

Exploring junctional complexes in genetic model organisms such as *Drosophila* and *C. elegans* offers several advantages and invaluable insights into the conservation of critical proteins across species. Despite variations in protein structure and organization, key proteins like cadherin, catenin, and claudin are well conserved in intestinal junctions across diverse organisms, highlighting their pivotal role in establishing a robust barrier system essential for intestinal protection, digestion, and immune function.^49^ In addition, model organisms often feature rapid life cycles, large broods, and sophisticated genetic toolkits, enabling the genetic study of intestinal junctional complexes *in vivo* with unparalleled efficiency. Moreover, the transparent bodies of *C. elegans* and *Drosophila* larvae facilitate direct visual assessment of gut leakage, enhancing our ability to study intestinal barrier function.^50,51^ Furthermore, *Drosophila* and *C. elegans* are ideal for high-throughput genetic and chemical screens. Researchers can quickly test the effects of many genes or compounds on intestinal barrier function, aiding in the discovery of potential therapeutic targets and drugs.

While *D. melanogaster* and *C. elegans* offer significant advantages for understanding basic and evolutionary aspects of intestinal barriers, using these invertebrate models to study human intestinal diseases presents several limitations. For example, the macro-structure of the intestine and signaling pathways can significantly differ among mammals, nematodes, and insects.^52^ Moreover, *D. melanogaster* and *C. elegans* lack an adaptive immune response, thus missing key cell types and cellular processes integral to mammalian intestinal epithelium.^52^ Additionally, the composition of gut microbes differs drastically between invertebrate models and humans, with *D. melanogaster* and *C. elegans* harboring much simpler and less diverse microbial communities tailored to their evolutionary and physiological needs.^52^ Thus, to better mimic human intestinal diseases such as IBDs, rodent models are often preferred to mitigate these limitations.^53^

Mice have a GI tract anatomically similar to humans, with analogous elements of adaptive immune response.^52^ The gut microbiome in mice is also similar in composition to humans with the same species diversity of bacteria phyla.^52^ Genetically, mice are invaluable for studying specific genes due to their high homology with humans and the ability to generate specific genetic knockouts. Various methods induce IBD-like symptoms in mice, such as trinitrobenzene sulfonic acid (TNBS) treatment in the BALB/c background to mimic Crohn’s disease-like symptoms, whereas C57BL/6 mice are relatively unaffected.^52,53^ Another common model is sodium dextran sulfate (DSS)-induced colitis, which induces inflammation and ulcerative colitis symptoms, with severity varying across different mouse strains.^52,53^ The robustness of murine systems in modeling IBDs has been pivotal for advancing research in this field. Many modern discoveries in IBD treatment and management stem from foundational studies in mice. For a comprehensive discussion on the advantages and disadvantages of various model organisms and their contributions to understanding IBDs and related intestinal conditions, please refer to insightful reviews by Jiminez et al. and Kiesler et al., which provide extensive coverage of this topic.^52,53^

## 3. Techniques for assessing intestinal permeability

Due to the importance of intestinal barrier integrity, numerous experimental procedures have been developed to measure intestinal permeability both *in vitro* and *in vivo*. Here we highlight a selection of the most widespread methods for assaying gut leakage.

### 3.1. Oral probe excretion assays

**Figure 2.**
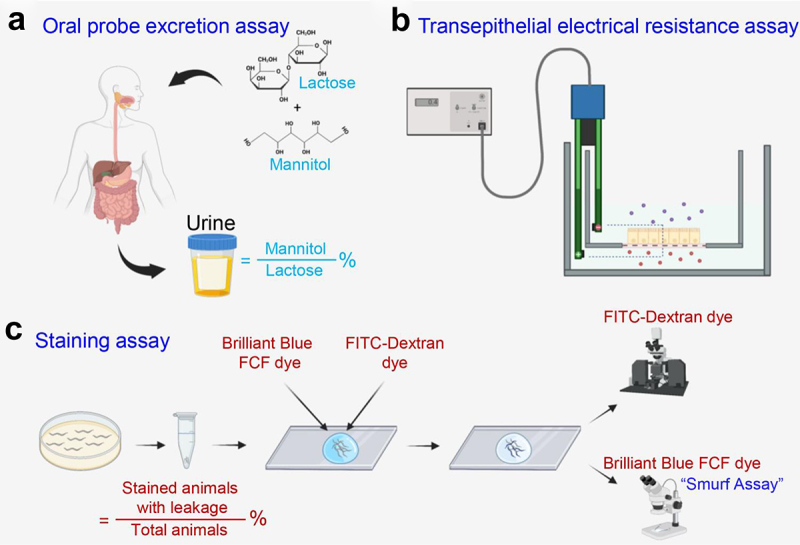
Popular methods for assessing intestinal permeability. (a), the oral probe excretion assay is a widely employed indirect method where probe molecules traverse the gastrointestinal system and potentially cross the intestinal barrier, subsequently quantified through renal excretion assays to gauge intestinal permeability. (b), the transepithelial electrical resistance (TEER) assay measures the electrical resistance across a cell monolayer, commonly used to assess the integrity and barrier function of epithelial or endothelial cell layers. (c), the gut permeability staining assay involves the visualization of fluorescently labeled molecules or tracers that permeate through the gut epithelium, thus providing insights into intestinal barrier integrity and permeability. This method is commonly used in model organisms with transparent anatomy.

One of the more traditional methods for measuring human intestine permeability is through administration of saccharide-like or radiolabeled probe molecules. These probes are chosen for specific properties such as their resistance to bacterial degradation, solubility in water, and relative inertness.^54^ Also key to these probes is their inability to pass through the intestinal barrier via transcellular pathways.^55^ Following ingestion, the probe molecules pass through the GI system where they may leak across the intestinal barrier. The leakage can then be quantified via renal excretion assays and serve as a marker for intestine permeability (Figure 2(a)). Depending on their sizes and the severity of leakage in gastric tissues, different ratios of the probes can be detected in the urine of subjects.^56^ For example, the monosaccharide probe mannitol is relatively small with a diameter of 6.5 Å and believed to cross the epithelial barrier through the pore pathway.^54^ In contrast, the disaccharide probe lactulose is a larger molecule with the diameter of 9.5 Å and thought to only cross via the leak pathway.^54^ The lactulose:mannitol ratio (LMR) in urine samples can therefore provide insight on the activity of these two pathways. Studies have reported a LMR in healthy individuals to be 0.014, while in IBD patients the LMR increases 10-fold to 0.093–0.133.^57^ Intriguingly, healthy individuals who are first-degree relatives of IBD patients often exhibit elevated LMRs prior to disease onset and have a markedly increased predisposition to developing IBD later in life.^19^

While the use of oral probes in detecting intestinal permeability is convenient, it is important to note its complications. First, oral probes are indirect measurement of intestinal permeability. Second, recent research has challenged the accuracy of oral probes in measuring gut permeability. For example, the sizes of two popular oral probes mannitol and lactulose may not differ significantly.^58^ However, mannitol is excreted in orders-of-magnitude higher concentrations than lactulose: 31.2 ± 3.4% of administered ^13^C-mannitol is excreted within 24 hours in healthy adults, compared to just 0.32 ± 0.03% for lactulose.^59^ Thus, the evidence for similarly-sized probes with differing excretion rates questions the validity of the leak versus pore distinction since both molecules may be crossing via the same pathway. Taken together, cautions need to be taken to interpret the result of using oral probes to detect leaky gut.

### 3.2. Transepithelial electrical resistance assay and Ussing chamber assay

The Transepithelial Electrical Resistance (TEER) assay is an *in vitro* method for real time and direct measurements of barrier integrity in cellular monolayers.^60^ TEER is frequently used to study intestinal tissue permeability in Caco-2 cells derived from a human colorectal adenocarcinoma, but is also useful for other, intact epithelial tissues such as the pulmonary alveolar epithelial barrier, urinary tract epithelial barrier, and intestinal epithelial barrier.^61,62^ Caco-2 cells grow naturally into monolayers and have many similar functions to *in vivo* small intestine villi epithelium.^63^

For grown cellular monolayers, the TEER assay involves culturing the tissue of interest as a single-cell layer on a semi-permeable membrane, forming a tight monolayer that mimics the epithelial barrier *in vivo*. With electrodes placed in the apical and basolateral compartments, a small electrical current is applied across the cell monolayer, and the resistance to this current is measured (Figure 2(b)).^64,65^

TEER measurements exhibit considerable variability due to factors such as cell culture conditions, experimental setup, and the specific laboratory conducting the assay. Typically, data are presented as unit area resistance, calculated by dividing resistance values by the membrane area.^66^ For instance, TEER values in Caco-2 cells have been documented to range from 49 to 137 Ω/cm^2^ depending on their degree of differentiation.^61^ Additionally, studies have reported TEER measurements as high as nearly 400 Ω/cm^2^ in healthy Caco-2 cell controls, while exposure to lipopolysaccharide (LPS) can lead to a decrease of up to 50% in TEER values.^66^ For an extensive compilation of TEER values in Caco-2 monolayers across various experimental setups, refer to Srinivasan et al.^60^

When studying whole-tissue samples like the intestine, researchers often utilize the Ussing chamber assay. This method preserves the heterogeneity and morphology of intact intestinal tissue by mounting it in a specialized chamber that allows separate solutions on both apical and basolateral sides.^62^ Unlike the TEER assay, which measures the resistance of cellular monolayers, the Ussing chamber assay evaluates the electrical properties such as voltage and the movement of ions across the epithelial barrier under controlled conditions. Combining the Ussing chamber with radio-labeled probes like^14^C and^3^H allows for quantitative assessment of barrier permeability.^67^ This approach provides valuable insights into ion transport, secretion, and absorption across epithelial tissues *ex vivo*.

### 3.3. Staining assays

In addition to the methods outlined above, the Smurf assay and Fluorescein isothiocyanate (FITC)-Dextran staining assay are commonly used to visualize leaky gut. The Smurf assay involves feeding a non-absorbable blue dye to animals and monitoring the leakage of the dye from the intestine into the body (Figure 2(c)).^51^ The most frequently used dye is FD&C Blue No. 1 (disodium 2-[[4-[ethyl-[(3-sulfonatophenyl)-methyl]amino]phenyl]-[4-[ethyl-[(3-sulfonatophenyl)methyl]-azaniumylidene]cyclohexa-2,5-dien-1-ylidene]methyl]benzenesulfonate) which is more commonly known as Brilliant Blue FCF. This dye has been widely used in commercially processed foods as a coloring agent since it was first approved by the FDA in 1993.^68^

The Smurf assay, pioneered in *D. melanogaster* and later adapted for *C. elegans*, allows for the identification of ‘blue Smurfs’ – organisms exhibiting increased intestinal leakage, indicated by a blue-stained appearance.^51^ Following dye ingestion and an incubation period, animals can be sorted based on the color of their bodies, either normally colored or stained blue. Animals with blue bodies are a result of increased leakage from the intestine. This leakiness leads to the characteristic blue stained appearance by which the assay gets its name, i.e. blue Smurfs.^51,69^ Notably, the Smurf phenotype is typically assessed as a binary outcome, reflecting either increased permeability or an intact barrier. The proportion of Smurf animals within a population serves as a measure of overall leakiness and has been linked to lifespan in various organisms.^69^ Importantly, studies have demonstrated a correlation between the proportion of ‘blue Smurfs’ and lifespan. In *Drosophila*, the proportion of wildtype flies exhibiting the Smurf phenotype increases with age.^51^ Conversely, the *eat-2* mutant of *C. elegans*, known for its extended lifespan due to dietary restriction, displays a markedly lower Smurf proportion compared to wildtype counterparts.^70^ These findings align with observations in humans, indicating that age plays a pivotal role in determining intestinal permeability.^71^

Another method for evaluating intestinal permeability is the FITC-Dextran staining assay.^72^ Operating on a principles akin to the Smurf assay, this method may be performed by orally administering fluorescently labeled FITC-Dextran to animals or by staining *in vitro* cellular monolayers and three-dimensional animal and human intestinal organoids.^73^ Subsequent monitoring allows detection of leakage from the tissue of interest into the surrounding tissue (Figure 2(c)), serving as an indicator of permeability. Enhanced fluorescence observed in across the barrier tissue corresponds with heightened levels of intestinal leakage and compromised integrity of junction protein complexes.^66^ It is important to note that the commonly used FITC-4 kDa dextran probe for assessing intestinal permeability cannot differentiate between leak and unrestricted pathways, as 4 kDa dextran can cross both. Additionally, 4 kDa dextran is too large to pass through the pore pathway, thereby providing no information about paracellular flux through this route. To quantitatively measure barrier permeability across pore, leak, and unrestricted pathways, and to accurately assess flux changes due to defects in cell junctions or overall epithelial damage, a three-probe fluorescent system has been developed.^74^ This assay utilizes three separate probes: creatinine (6 Å diameter), FITC-4 kDa dextran (28 Å diameter), and rhodamine-70 kDa dextran (120 Å diameter), each probing different leakage routes. Creatinine can permeate all three pathways, while 4 kDa dextran is restricted to leak and unrestricted pathways, and 70 kDa dextran is limited to the unrestricted pathway.^74^ Importantly, this technique can be applied either *in vivo* via oral gavage or *ex vivo* using the Ussing chamber, thereby combining the advantages of both approaches.

## 4. Intrinsic factors involved in regulating intestinal barrier complexes

**Figure 3.**
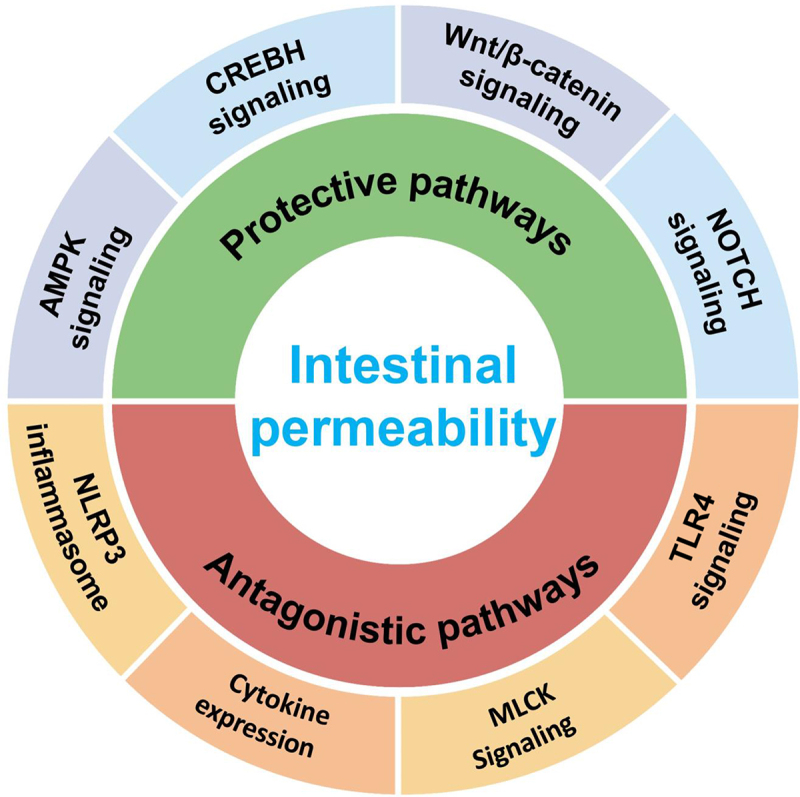
A summary scheme of the intrinsic cellular signaling pathways modulating intestinal permeability.

**Figure 4.**
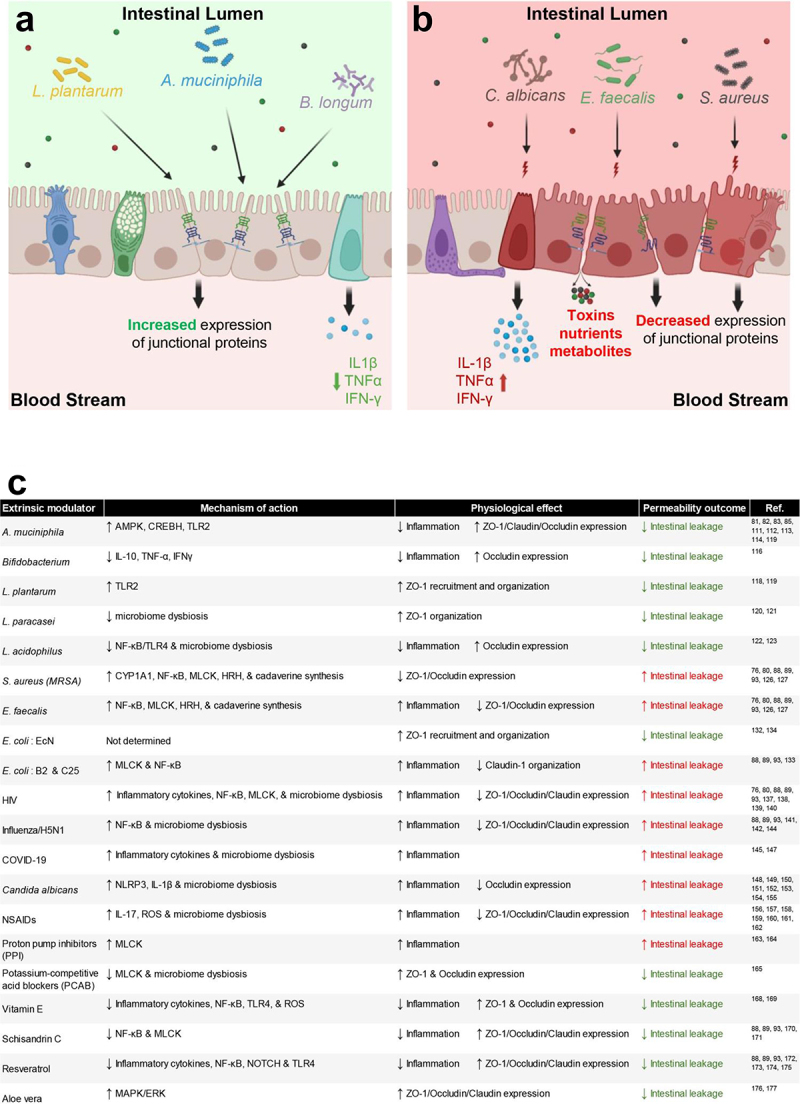
Gut microbes and common chemicals regulate intestinal junctional complexes in a complex manner. (a), many beneficial microbes (e.g., *L. plantarum*, *A. muciniphila*, *B. longum*) promote the gut barrier integrity and reduce cytokine release and inflammation. (b), detrimental microbes (e.g., *C. albicans*, *E. faecalis*, *S. aureus*) undermine gut barrier integrity by diminishing junctional protein expression and organization. (c), a summary of microbes and chemicals that are known to affect gut barrier permeability through diverse mechanisms.

As previously described, alterations in intestinal permeability often precede the onset of many intestinal and extraintestinal disorders. Thus, unraveling the factors that contribute to changes in intestine permeability has become an important facet in combating these diseases. In the subsequent sections, we focus on modulators that impact the integrity of the intestinal barrier complexes, highlighting endogenous (Figure 3) and exogenous (Figure 4) contributors to intestinal leakage. Herein, we present a compilation of signaling pathways and effectors widely implicated in GI diseases, most of which have been predominantly studied using mouse models of intestinal barriers. While this list is not exhaustive, it highlights some of the most recognized and influential pathways known to regulate the intestinal epithelial barrier.

### 4.1. Myosin light chain kinase (MLCK) pathway

Among the myriad signaling pathways implicated in the regulation of gut barrier complexes, the myosin light chain kinase (MLCK) stands out as a primary player. Encoded by the MYLK1 gene, intestinal MLCK exists in two splice variants: MLCK1 and MLCK2.^75^ Both variants are calmodulin-activated serine/threonine kinases long known to orchestrate tight junction organization in intestinal epithelial cells, a process intricately linked to Na^+^-glucose cotransport.^75^ Mechanistically, the demand for Na^+^ transport across the barrier triggers MLCK activation, leading to the phosphorylation of myosin II regulatory light chain (MLC) within the perijunctional actomyosin ring (PAMR). This phosphorylation triggers a contractile force that increases physical tension on tight junctions, potentially causing structural deformations and thereby elevating paracellular permeability.^76^ Enhanced expression and activity of MLCK are frequently observed in cases of IBDs in both rodent models and human patients, making it an attractive therapeutic target.^77,78^ Several recent studies have explored the potential of MLCK inhibition in controlling IBD pathology and reducing intestinal permeability.^79,80^

In a recent study, researchers demonstrated that recruitment of MLCK1 to the PAMR, followed by MLC phosphorylation, heightened permeability, as evidenced by a reduction in TEER.^80^ Through screening a library of 140,000 compounds for MLCK1 inhibitory activity, they identified a novel compound termed “divertin”, which impedes the recruitment of MLCK1 to the PAMR. Addition of divertin to TNF-treated cells restored TEER levels to those of healthy, non-TNF-treated cells and reversed MLC phosphorylation.^80^ These findings were corroborated *in vivo*, as divertin protected mice administered TNF to induce barrier dysfunction. In the absence of MLCK1, MLC phosphorylation is inhibited, preventing occludin endocytosis and thereby preserving tight junctions integrity.^80^

### 4.2. AMP-activated protein kinase (AMPK) pathway

AMPK signaling has also been implicated as a key pathway for regulating inflammation and tight junction expression within the intestine. In a DSS-induced colitis model, genetic deletion of AMPK exacerbated the severity of IBD symptoms, potentially attributed to heightened release of proinflammatory cytokines and increased macrophage activity.^81^ Conversely, activation of AMPK directly influences tight junctions assembly. For instance, treatment with AICAR, a nonspecific AMPK activator, accelerates the recruitment of ZO-1 protein to the membrane, reduces permeability to FITC-dextran, enhances TEER, and upregulates markers indicative of intestinal differentiation.^82^ Notably, the transcription factor CDX2, crucial for cell differentiation, is upregulated following AMPK activation. Inversely, inhibition of AMPK abolished the protective permeability effects seen and CRISPR/Cas9 deletion of CDX2 abolished differentiation.^82^ Overall, AMPK serves as a critical modulator of intestinal permeability and tight junction assembly, likely mediated through mechanisms involving cellular differentiation and recruitment of tight junction proteins to the membrane.^83^

### 4.3. cAMP-responsive element-binding protein H (CREBH) pathway

The cAMP signaling pathway has been extensively explored for its impact on intestinal permeability modulation. Central to this pathway is the cAMP-responsive element-binding protein H (CREBH), acting as a pivotal transcription factor.^84^ Given reports of defective cAMP signaling in pediatric colitis and the presence of CREBH in intestinal epithelial cells, it was postulated that they play a pivotal role in regulating intestinal permeability.^85^ In a mouse model of DSS-induced colitis, both CREBH mRNA and protein expression were diminished.^85^ Notably, both DSS-treated wild type mice and CREBH knockout mice exhibited decreased expression of tight junction proteins critical for maintaining intestinal barrier integrity, including claudin-1, claudin-3, claudin-5, claudin-8, and ZO-1.^85^ Intriguingly, there was also a substantial increase in the expression of claudin-2, a known mediator of leaky gut and promoter of IBD progression.^85^ This regulation of tight junction proteins via CREBH may be related to IGF signaling, which is known to stimulate epithelial cell proliferation following injuries to the intestine.^86^ Further investigations unveiled a downregulation of IGF1R in both DSS-treated wild type mice and CREBH knockout mice, with expression levels being restorable through forced expression of CREBH.^85^ Together, these findings suggest that CREBH plays a crucial role in stimulating IGF1R expression, ultimately leading to an increase in the expression of essential tight junction proteins. This mechanism contributes to the enhancement of gut barrier health and the reduction of permeability, offering potential therapeutic avenues for addressing intestinal disorders.

### 4.4. TLR4 pathway

The Toll-like receptor 4 (TLR4) signaling pathway stands as a cornerstone of innate immunity, and it is a pivotal signaling pathway governing gut permeability through its activation of downstream proinflammatory signaling events. Specifically, TLR4 serves as a pivotal receptor known for its recognition of bacterial remnants, notably LPS.^87^ Within the intestine, LPS engagement with epithelial TLR4 receptors initiates a signaling cascade culminating in the activation of nuclear factor–κB (NF-κB) transcription factors and other proinflammatory processes.^88,89^ Elevated NF-κBp65 levels and intestinal inflammation have been associated with decreased expression of ZO-1 and occludin.^90^

While basal TLR4 expression in intestinal tissue is low, it plays a crucial role in safeguarding against intestinal and bacterial injuries.^88,91^ Notably, studies have consistently observed elevated TLR4 expression in the intestinal cells of individuals with IBD, with overexpression in murine models correlating with increased susceptibility to chemically induced colitis.^87,92^ Moreover, experiments with TLR4-overexpressing mice have demonstrated compromised intestinal barrier function, leading to heightened permeability and leakage of FITC-dextran into the serum.^93^

### 4.5. Wnt/β-catenin pathway

The canonical Wnt signaling pathway, also referred to as the Wnt/β-catenin pathway, plays a pivotal role in cell proliferation by stabilizing and translocating β-catenin into the nucleus.^94^ In the intestine, this pathway is indispensable for the maintenance and regeneration of intestinal stem cells and tissue integrity.^95^ Dysregulation of Wnt signaling is implicated in various intestinal diseases, including necrotizing enterocolitis and IBDs, which are characterized by intestinal injury, inflammation, and compromised gut barrier function.^95,96^

Activation of Wnt signaling leads to the nuclear translocation of β-catenin, which then activates many target genes through β-catenin-T-cell factor/lymphoid enhancer-binding factor (TCF/LEF) transcription factors. Notably, these target genes include proteins crucial for tight junction assembly in the intestine, such as ZO-1 and occludin.^90^ Studies in mice have shown that disruption of Wnt/β-catenin signaling results in reduced mRNA expression of ZO-1 and occludin, accompanied by increased intestinal permeability, as indicated by FITC-dextran staining. Conversely, restoration of Wnt signaling reverses these effects, highlighting the importance of Wnt signaling in maintaining intestinal barrier function.^90^ Furthermore, Wnt signaling has been found to inhibit NF-κB activity through direct interaction of β-catenin with NF-κB, thereby mitigating inflammation and tissue damage.^97,98^ Together, these results implicate *Wnt*/β-catenin signaling as another important pathway affecting intestinal inflammation and junctional organization.

### 4.6. Notch pathway

NOTCH receptors also play a crucial role in regulating the integrity of the intestinal barrier. These membrane-bound proteins undergo endocytosis and nuclear translocation upon activation by their binding partners.^99^ Once cleaved in the nucleus, NOTCH acts as a transcription factor, orchestrating the expression of numerous genes involved in development, tissue repair, and cell differentiation.^99^ In the GI tract, NOTCH activity is specifically linked to the regulation of tight junction proteins. Interestingly, in mice lacking lamina propria lymphocytes that induce intestinal epithelial differentiation, the absence of cleaved Notch-1 protein correlated with increased intestinal permeability. This was evidenced by reduced TEER measurements and increased FITC-dextran staining.^100^ Further evidence from Notch-1 knockdown experiments in Caco-2 cell lines underscores NOTCH’s direct impact on barrier integrity, with increased intestinal permeability observed compared to control lines.^100^ These studies suggest that cleaved NOTCH proteins transcriptionally regulate the expression of proteins crucial for the architecture of intestinal junction complexes.^100^

While the relationship between NOTCH activity and specific junction proteins may vary, research indicates a direct correlation between NOTCH activity and the expression of proteins such as occludin and claudin-1, while an inverse relationship exists with proteins like claudin-5.^100,101^ Overall, the NOTCH pathway emerges as another critical signaling pathway intricately linked to the expression and maintenance of junction complex proteins in the intestinal epithelial barrier.

### 4.7. Autophagy

Routine cellular processes such as autophagy, along with other stress-response factors, play a pivotal role in regulating tight junction architecture and, consequently, are essential for maintaining barrier integrity. Autophagy is a highly conserved mechanism of cellular recycling that eliminates damaged and aged proteins.^102^ This homeostatic process is crucial for responding to cellular stress and is particularly active in the proliferative component of colonic crypts.^103^ Under stress conditions, the autophagic machinery engulfs cytoplasmic components, which are subsequently degraded upon fusion with lysosomes.^103^

Regarding its impact on permeability, autophagy induction protects the intestinal barrier by influencing the expression of tight junction proteins. For instance, claudin-2, a pore-forming claudin protein, undergoes lysosomal degradation during starvation-induced autophagy.^102^ Conversely, TNF-mediated inhibition of autophagy leads to increased claudin-2 expression and heightened permeability.^103^ Beyond claudin-2, autophagic activity also impedes the endocytosis of occludin protein, thereby preserving tight junction structure.^104^

## 5. Microbes involved in regulating intestinal barrier complexes

The gut microbiome constitutes a complex ecosystem of microorganisms inhabiting the human intestinal epithelium. This ecosystem undergoes dynamic changes throughout life, with newborns initially possessing a sterile intestinal tract. However, by adulthood, the gut microbiome can comprise up to 10^14^ cells, greatly outnumbering their own host cells nearly tenfold.^105^ Among these microorganisms, bacteria predominate, though the microbiome also encompasses fungi, viruses, and other organisms, with estimates suggesting the presence of 300 to 1000 bacterial species.^106,107^ While many microbes are pathogenic and cause damage to the epithelium, countless others have been investigated for their role as probiotics, improving the health and integrity of the intestine. The gut microecosystem maintains a delicate balance between beneficial and pathogenic microbes, and disruptions to this balance can lead to dysbiosis and the overgrowth of opportunistic organisms. In healthy individuals, beneficial microbes effectively outcompete specific pathogenic strains, thereby limiting their ability to spread and cause infections.^108^ In contrast, patients suffering from IBDs and other related conditions often exhibit a disturbed microbiome that exacerbates inflammation and compromises barrier function.^109^ Although these observations are now recognized as hallmarks of IBD pathology, whether they are causes and/or effects of the disease is still debated.^109^ Understanding how microbiome alterations influence IBDs and contribute to intestinal barrier function is essential for developing effective treatment strategies for these diseases. Due to the open questions that remain in this field of research, numerous studies over the past decade have highlighted the profound impact of bacterial colonization on gut permeability (Figure 4).

### 5.1. Beneficial microbes for intestinal barrier complexes

Of particular interest is the utilization of probiotics to mitigate gut inflammation and subsequent permeability. Probiotics often exert their beneficial effects on permeability by modulating proteins within cell-adhesion junctions. For example, *Akkermansia muciniphila* (*A. muciniphila*) and its associated compounds have demonstrated efficacy in reducing intestinal inflammation and permeability in both *in vitro* and *in vivo* models through diverse molecular pathways (Figure 4(a)).

High-fat diets (HFD) are recognized for their propensity to increase permeability in experimental colitis models and exacerbate symptoms of IBD in humans.^110^ HFDs reduce the expression of tight junction components such as ZO-1 and occludin, compromising gut barrier integrity and elevating overall permeability.^111^ Interestingly, HFD-fed mice administered *A. muciniphila*-derived extracellular vesicles (AmEVs) exhibited a marked reduction in permeability compared to those on the HFD alone.^112^ Notably, AmEV-treated mice displayed elevated expression of tight junction proteins like occludin and claudin, countering the detrimental effects of the HFD.^112^ This suggests that barrier reinforcement is associated with enhanced cellular connectivity within the intestine.^113^ Mechanistically, the upregulation of tight junction proteins induced by AmEVs appears to involve multiple pathways including the aforementioned phosphorylation of AMPK and cAMP signaling via CREBH (Figure 4(c)).^85,112^ Consequently, it is unsurprising that treatment of Caco-2 cells with AmEVs resulted in elevated AMPK phosphorylation, accompanied by a correlated reduction in intestinal permeability. By contrast, pharmacological inhibition of AMPK in AmEV-treated cells attenuated the protective effects observed in previous experiments.^112^ In addition to AMPK, *A. muciniphila* may also be exerting its effect through the activation of CREBH. Infection of Caco-2 cells with *A. muciniphila* resulted in the upregulation of CREBH expression, leading to the mitigation of gut leakage.^85^ This effect may be attributed to the presence of Amuc_1100, an abundant outer membrane protein of *A. muciniphila* known to regulate CREBH expression.^85^ Furthermore, Amuc_1100 was shown to upregulate the expression of toll-like receptor 2 (TLR2), a key player in intestinal wound healing.^85,114^ Taken together, these findings suggest that *A. muciniphila* and its protein components confer a protective effect on intestinal barrier integrity, potentially through modulation of multiple signaling pathways.

The supplementation of human diets with gram-positive, lactic-acid producing bacteria (LAB) has long been recognized for its beneficial effects in combating various human diseases.^115^ Among these bacteria, multiple species of the *Bifidobacterium* genus, commonly found in the human gut microbiome, have shown promise in regulating proinflammatory cytokine expression and promoting epithelial barrier function. Studies have revealed that *Bifidobacterium longum ssp. longum* can downregulate the expression of proinflammatory markers TNF-α and IFN-γ, along with the anti-inflammatory cytokine IL-10, indicating a reduced inflammatory state and decreased demand for anti-inflammatory responses (Figure 4(c)).^116^ This bacterial strain has also demonstrated the ability to decrease permeability as assessed by FITC-dextran measurements in a DSS-induced colitis mouse model.^116^ Likewise, *Bifidobacterium bifidum* (*B. bifidum*) has demonstrated beneficial effects on barrier integrity. In experiments using Caco-2 cell monolayers treated with TNF-α to induce a decrease in TEER, concurrent administration of *B. bifidum* effectively restores TEER levels and enhances occludin expression.^117^

*Lactobacillus*, another genus of LAB prevalent in the human intestine, has been shown to modulate intestinal permeability. In a randomized controlled trial involving healthy human subjects, administration of *Lactobacillus plantarum* (*L. plantarum*) directly to the duodenum resulted in alterations in the expression pattern of the tight junction protein ZO-1.^118^ Interestingly, the presence of *L. plantarum* appears to force apical localization of ZO-1 and increase tight junction presence on cell surface membranes.^118,119^ While this increase in tight junction presence alone did not enhance barrier integrity in a Caco-2 monolayer, *L. plantarum* colonization significantly attenuated the increase in permeability induced by phorbol 12,13-dibutyrate (PDBu), a derivative of TPA known to cause dislocation of tight junction proteins.^118^ Previous research has demonstrated that administration of Pam3-Cys-SK4 (PCSK), an artificial Toll-like receptor 2 (TLR2) ligand, restored tight junction integrity in DSS-induced colitis mice.^119^ Given evidence suggesting that TLR2 agonism increases translocation of ZO-1 to tight junctions and confers protection, researchers investigated whether the effects of *L. plantarum* are mediated by TLR2 signaling. By assessing TEER and employing phorbol treatment, they found that activating TLR2 with PCSK replicated the effects observed with *L. plantarum* treatment alone.^118^ These findings imply that changes in ZO-1 localization induced by *L. plantarum* may indeed be mediated by TLR2 signaling (Figure 4(c)).

*Lactobacillus paracasei* (*L. paracasei*) and *Lactobacillus acidophilus* (*L. acidophilus*), two other species within the *Lactobacillus* genus, have also demonstrated protective effects against epithelial barrier damage.^120^ *Salmonella typhimurium (S. typhimurium)*, a pathogenic bacterial strain known for inducing intestinal epithelial destruction and irregular remodeling of tight junction proteins, poses a significant threat to gut health.^121^ However, a recent study revealed that a metabolite derived from *L. paracasei* effectively counteracts the damaging effects of *S. typhimurium*.^120^ Specifically, *L. paracasei* CNCM I-5220-derived postbiotic (LP-PBF) was found to prevent the disorganization of ZO-1 in tight junctions and increase TEER values in cell monolayers; remarkably, these protective effects were achieved without adversely affecting the commensal gut microbiota.^120^ Unlike antibiotics, which target bacterial growth, LP-PBF appears to neutralize *S. typhimurium* and restrict its ability to invade the intestinal epithelium, possibly by impeding the formation of *S. typhimurium* biofilms.^120^ Unlike *L. paracasei*, *L. acidophilus* exerts its protective function by directly modulating the expression of tight junction proteins. As early as 2005, studies demonstrated that treating rats with a probiotic cocktail containing *L. acidophilus* increased the expression of occludin.^122^ More recent research has shown that mice fed high-fat diets and subsequently administered fecal transplants of *L. acidophilus* experienced reduced inflammation and microbiome dysbiosis. Similar to the effect observed with *A. muciniphila*, these mice also showed improved permeability outcomes, indicated by decreased FITC-dextran staining and increased occludin expression.^123^ Improved barrier integrity is attributed to decreased activation of the TLR4 and NF-κB signaling pathways, as well as reduced expression of downstream proinflammatory cytokines.^123^ Numerous studies on *L. plantarum*, *L. paracasei*, and *L. acidophilus* collectively underscore the critical role of the *Lactobacillus* genus in maintaining gut barrier integrity.

### 5.2. Detrimental microbes for intestinal barrier complexes

While approximately 93% of known bacterial species are deemed nonpathogenic, the gut harbors a diverse range of microbes, some of which can cause diseases.^124^ Among these, *Staphylococcus aureus* (*S. aureus*), a gram-negative bacterium commonly found in the human nasal mucosa, poses a significant health risk.^125^ While *S. aureus* infections are prevalent on the skin, they become especially dangerous when they penetrate deeper tissues, leading to conditions like sepsis and organ failure, particularly when the strains are antibiotic-resistant (methicillin-resistant *S. aureus*, or MRSA) (Figure 4(b)).^124,125^

MRSA-induced sepsis can also have profoundly negative effects on gut barrier function, possibly due to increased expression of cytochrome P4501A1 (CYP1A1) (Figure 4(c)). In patients with sepsis, increased levels of CYP1A1 have been documented and CYP1A1 inhibitors exhibit anti-inflammatory, antitumor, and other protective immune functions.^126,127^ Similarly, studies involving mouse models of MRSA infection have shown that mice lacking CYP1A1 have a much higher survival rate and better preservation of gut barrier proteins (e.g., ZO-1, occludin, and E-cadherin) following MRSA infection compared to wildtype mice.^127^ Further investigations revealed the involvement of cadaverine, a byproduct of lysine metabolism produced by gut microflora, in MRSA-induced gut permeability.^127^ Cadaverine levels rise significantly during MRSA infection, with CYP1A1 potentially playing a crucial role in its synthesis.^127^ Interestingly, pretreatment with cadaverine abolishes the protective effects against MRSA infection in *Cyp1a1* knockout mice, indicating the importance of CYP1A1 in this context.^127^

Among the various commensal gut microbiota, *Enterococcus faecalis* (*E. faecalis*) stands out as one of the most prolific producers of cadaverine. In the cecal contents of both *Cyp1a1*^*+/+*^ and *Cyp1a1*^*–/–*^ mice, cadaverine levels were increased following oral gavage with *E. faecalis*.^127^ Co-infection of *Cyp1a1*^*+/+*^ mice with *E. faecalis* and MRSA leads to notable outcomes, including a decrease in ZO-1 expression, heightened intestinal permeability, and reduced survivability compared to untreated *Cyp1a1*^*–/–*^ mice. Interestingly, *Cyp1a1* knockouts maintain a protective phenotype against these effects, indicating the crucial role of CYP1A1 in gut homeostasis.^127^ Mechanistically, the increased activities in MLCK and NF-κB pathways are likely main drivers behind the increase in intestine permeability, as cadaverine can cause downstream activation of both MLCK and NF-κB via agonism of histamine receptor (HRH) family member HRH4 (Figure 4(c)).^127^ Notably, HRH4 expression is also upregulated in the epithelium of *Cyp1a1*^*+/+*^ mice with MRSA infection.^127^ These findings collectively underscore the contribution of both MRSA and *E. faecalis*—despite their natural presence in the human gut microbiome – to the pathology of leaky gut. Their induction of cadaverine levels, leading to NF-κB pathway activation, highlights the intricate interplay between gut microbiota and intestinal barrier integrity.

Interestingly, despite *Cyp1a1*^*-/-*^ mice being protected against MRSA infection, *Cyp1a1* expression does not consistently indicate permeability status. The aryl-hydrocarbon receptor (AhR) is a ligand-activated transcription factor that detects xenobiotic compounds and regulates genes involved in xenobiotic metabolism, including *Cyp1a1*.^128^ Activation of AhR by the microbial metabolite Urolithin A (UroA) mitigated symptoms of DSS-induced colitis in mice, but this effect was absent in *Cyp1a1-/-* animals.^129^ Furthermore, wildtype mice treated with UroA showed reduced FITC-dextran staining and significant increases in ZO-1, occludin, and claudin-4, whereas *Cyp1a1-/-* mice did not exhibit improvement.^129^ This suggests that while AhR activation by certain immune pathways can protect barrier integrity, prolonged activation (as seen in MRSA infection) may impair the intestinal epithelium barrier.^130^

AhR is activated by a variety of tryptophan-based ligands such as indole, indole-3-acetic acid, and indole-3-aldehyde, all of which are derived from indigenous microbiome members.^131^ The role of AhR as a regulator of intestinal barrier function is underscored by its responsiveness to diverse microbial-derived ligands. Ongoing research into the generation of AhR ligands by the microbiota will further clarify the influence of this receptor on intestinal permeability.^131^

### 5.3. Context-dependent bacterial modulators of the intestinal barrier complexes

Not all bacteria species follow the binary classification of either promoting or suppressing leaky gut pathology. For example, some evidence suggests that gut colonization with *Escherichia coli* (*E. coli*) may reduce permeability while other studies report an increase in permeability, dependent on the specific strain of *E. coli*.^132,133^ *E. coli Nissle 1917 (EcN)*, for instance, has been observed to increase ZO-1 expression in healthy and DSS-induced colitis mouse models (Figure 4(c)).^132^ While the precise mechanism by which *EcN* controls ZO-1 expression remains unclear, it is known that ZO-1 plays a crucial role in the formation of tight junctions, thereby maintaining intestinal barrier integrity.^134^

Conversely, other strains of *E. coli* have been reported to weaken barrier integrity through different mechanisms. For instance, *E. coli* of the B2 phylotype commonly found in human intestines may produce α-hemolysin (HlyA), a toxin known to induce intestinal leakage, which is often present in higher levels in individuals with ulcerative colitis.^133^ Moreover, infection of T84 cell monolayers with *E. coli* strain C25 reduced TEER measurements and activated NF-κB signaling.^135^ Collectively, these examples underscore the diverse effects that gut bacteria can exert on permeability and junction remodeling, even within the same species, highlighting the complexity of interactions between microbes and host intestinal barriers.

### 5.4. Viral modulators of the intestinal barrier complexes

While bacteria dominate the human gut microbiota, comprising roughly 90%, the remaining 10% encompasses a diverse array of viruses, fungi, archaea, and protozoa species.^136^ Beyond bacteria, certain viral species emerge as significant exogenous regulators of intestinal permeability. Viruses and viral particles wield considerable influence over gut permeability through interactions with resident bacterial populations. Viral infections can precipitate shifts in microbiome composition, thereby impacting intestinal integrity. Notably, individuals infected with Human Immunodeficiency Virus (HIV) often experience marked alterations in gut microbiota, characterized by a decline in beneficial bacteria such as *Bifidobacterium* and an increase in harmful bacteria like *Pseudomonas*.^137^ This dysbiosis, linked to HIV infection, may directly contribute to leaky gut pathology. Moreover, research spanning decades has illuminated potential pathways through which HIV infection exacerbates intestinal permeability. Studies from the late 1990s revealed that HIV-positive patients exhibit a 1.5 to 3.1-fold greater lactulose-to-mannitol ratio in their urine, indicative of heightened intestinal leakage.^138^ HIV infection prompts host immune cells to release proinflammatory cytokines, potentially increasing permeability. Notably, two HIV proteins, envelope protein ‘gp120’ and transactivator protein ‘Tat’, directly modulate tight junction protein architecture. For instance, one study reported decreased expression of ZO-1, occludin, and claudin proteins in HIV-1-infected cells, with similar effects observed following treatment with isolated gp120.^139^ The host response to viral insult may involve production of TNF-α, IL-6, and IL-8 cytokines and activation of NF-κB signaling which may contribute to the increased permeability.^139^ Similarly, Tat protein induces expression of IL-18 and activity of MLCK, culminating in reduced claudin-2/occludin expression and compromised barrier integrity.^140^ Collectively, these findings underscore the complex interplay among viral infections, gut microbiota dynamics, and the pathophysiology of leaky gut syndrome in HIV-infected individuals.^138^

Influenza, a virus affecting various avian and mammalian species, primarily targets the upper respiratory tract but also induces secondary effects in the lower GI tract. Experimental models of intranasal influenza infection in mice demonstrate significant consequences such as shortened colons, diarrhea, and upregulation of proinflammatory genes in the intestines.^141,142^ Remarkably, direct infection of the gastric mucosa (intragastric) does not replicate the effects of intranasal injection, resulting in minimal pathological injury to the mice and complete clearance of the infection within 72 hours.^142^ These observations support the hypothesis that viral infection in the upper respiratory tract may adversely affect the distal intestinal mucosa. Evaluation of intestinal permeability reveals increased FITC-flux across the intestinal lumen into the surrounding plasma upon intranasal influenza infection.^141^ It is hypothesized that this elevation in inflammation markers and subsequent leaky gut pathophysiology could stem from a reduction in available short-chain fatty acids (SCFAs) – critical molecules for intestinal homeostasis.^141^ SCFAs, including acetate and butyrate, are produced by gut bacteria through the fermentation of dietary fiber, conferring protection against gut injury.^143^ Influenza infections, akin to other viral infections discussed, alter the gut microbiome, potentially favoring harmful microbes over SCFA-producing bacteria. This microbiome shift, coupled with decreased SCFA levels, correlates with the observed intestinal damage in influenza-infected mice and can be mitigated by direct administration of SCFAs.^141^ Additionally, it’s noteworthy that the H5N1 subtype of avian influenza virus has been found to directly downregulate E-cadherin, occludin, claudin-1, and ZO-1 in the alveolar tissue of infected mice via activation of TAK1-Itch, an upstream activator of NF-κB signaling.^144^ Although this data originates from a peripheral tissue rather than the intestine, similar phenomena may occur in the intestine, contributing to the observed increase in intestinal permeability.^144^

More recent studies have documented instances of microbiota dysbiosis in patients infected with SARS-CoV-2 (COVID-19).^145^ Similarly, mice carrying COVID-19 exhibited reduced microbial diversity and significant alterations in intestinal epithelial composition.^145^ Notably, severely ill mice showed diminished Paneth cell numbers, along with abnormalities in granule placement and morphology, coupled with reduced gene expression of antimicrobial factors such as lysozyme and defensins.^145^ These changes to the Paneth cells are reminiscent of changes seen in human cases of IBDs. In addition to mouse studies, researchers analyzed stool samples from human COVID-19 patients to monitor *Faecalibacterium* bacterium presence. *Faecalibacterium* species, commonly found in the human gut, play an immunosupportive role and inhibit NF-κB activation and IL-8 production.^146^ Reduced *Faecalibacterium* diversity in COVID-19 patients correlated negatively with nosocomial bloodstream infection (nBSI), indicating potential bacterial translocation from the intestine to the bloodstream.^145^ Furthermore, COVID-19 infection’s impact on alveolar tissue has been extensively studied, revealing the release of various cytokines that ultimately downregulate tight junction protein expression.^147^ Although these findings originate from a different tissue, they suggest a plausible link between COVID-19 infection and disruption of gut barrier integrity via similar mechanisms. While research on COVID-19‘s effects on intestinal health and diversity remains in its early stages, current evidence indicates that severe viral infection can compromise intestinal barrier function, potentially exacerbated by antibiotic use.^145^

### 5.5. Fungal modulators of intestinal barrier complexes

*Candida albicans* (*C. albicans*), a commonly found fungal species in the healthy human gut microbiome, usually maintains a commensal relationship without causing harm under normal circumstances. However, when exposed to stress or changes in gut bacterial composition, *C. albicans* may proliferate unchecked by outcompeting commensal microbes, increasing the risk of infection.^148^ In a mouse model of DSS-induced colitis, supplementation with *C. albicans* has been found to worsen colitis severity and heighten intestinal barrier permeability.^149^ Moreover, co-infection with *C. albicans* and *Klebsiella pneumoniae* has been shown to cause extensive damage to the gut barrier.^150^ This damage is attributed to increased expression of proinflammatory cytokines and simultaneous reduction in occludin.^150^

The primary means through which *C. albicans* inflicts damage on the intestine are via the production of Candidalysin, a cytolytic peptide toxin secreted upon epithelial infection.^151,152^ Candidalysin accumulation induces intestinal inflammation by triggering IL-1β release and activating the NLRP3 inflammasome, culminating in direct tissue damage and pyroptosis-mediated cell death.^151^ The NLRP3 (NACHT, LRR, and PYD domains-containing protein 3) inflammasome pathway is a significant proinflammatory pathway that responds to stress signals arising from injury or microbial invasion.^151^ While there are conflicting reports on the precise effects of NLRP3 inflammasome signaling on exacerbating IBDs, colitis, and intestinal permeability, NLRP3 inflammasome activation is considered a critical step in the damage inflicted by *C. albicans* infection.^151,153,154^ Previous studies have demonstrated that specific inhibitors targeting NLRP3 and IL-1β lead to increased expression of tight junction proteins.^155^ Consequently, NLRP3 activation through candidalysin secretion may have the contrary effect of reducing tight junction protein expression, thereby exacerbating gut damage and increasing permeability.^151^

## 6. Common chemicals involved in regulating intestinal barrier complexes

Nonsteroidal anti-inflammatory drugs (NSAIDs) are among the most commonly used medications due to their effective anti-inflammatory, analgesic, and antipyretic properties.^156^ However, chronic use of NSAIDs such as aspirin, especially in high doses, has been associated with increased gut permeability and alterations in the gut microbiome.^157,158^ Studies have shown that discontinuing NSAID use can restore gut permeability and microbiome composition to normal levels.^159^ The primary cause of NSAID-induced permeability increases is thought to be their mechanism of action as cyclooxygenase (COX) enzyme inhibitors, particularly within the gastrointestinal tract. More recent research has demonstrated that NSAIDs can upregulate interleukin IL-17A mRNA, and antibody neutralization of IL-17A can mitigate barrier damage.^160^ Additionally, NSAIDs have been shown to uncouple mitochondrial oxidative phosphorylation and generate reactive oxygen species (ROS).^156^ Interestingly, the intestinal damage caused by NSAID use can be either exacerbated or mitigated by co-administration with other compounds. For instance, when NSAID-administered mice were also given gliadin, a component of wheat gluten implicated in the progression of celiac disease, intestinal permeability more than doubled compared to mice given NSAIDs alone, and quadrupled compared to untreated mice.^161^ Conversely, NSAID-induced barrier damage was ameliorated in rodents given simultaneous doses of revaprazan, a potassium-competitive acid blocker (PCAB). Revaprazan prevented increases in intestinal permeability by enhancing the expression of tight junction proteins such as occludin, claudin, and ZO-1, likely due to the inactivation of Rho-GTPase, MLC, and ERK signaling pathways.^162^

Proton pump inhibitors (PPIs) effectively treat gastro-esophageal reflux disease (GERD), but their long-term use has been associated with gut barrier damage and worsening symptoms of IBDs.^163^ A 2023 study by Nighot et al. demonstrated that prolonged PPI use decreased TEER in cell culture by activating MLCK and exacerbated colitis in mouse models.^164^ This finding, along with other concerns about chronic PPI use, has spurred interest in identifying alternative therapies such as PCABs. For example, the novel PCAB tegoprazan has shown promise in improving barrier function by addressing microbiome dysbiosis, promoting the growth of beneficial bacteria, and increasing the expression of occludin and ZO-1.^165^ Further development of PCABs for conditions like GERD holds the potential to offer alternative therapeutic strategies that are less detrimental to gut barrier integrity.

Selective serotonin reuptake inhibitors (SSRIs) are widely prescribed for various mental health conditions, particularly depression and anxiety disorders. Some research suggests that SSRIs may contribute to gastrointestinal symptoms such as diarrhea, constipation, and abdominal discomfort, which may indicate potential effects on gut barrier function.^166^ While the direct causal link between SSRIs and leaky gut syndrome is not definitively established, SSRIs are known to modulate the intestinal barrier through several mechanisms. By altering serotonin levels, SSRIs may affect the tight junctions between intestinal epithelial cells, potentially influencing intestinal permeability. Moreover, serotonin plays a role in shaping the gut microbiota composition, and changes in serotonin levels induced by SSRIs can disrupt this balance, thereby impacting intestinal barrier function.^167^ Additionally, serotonin receptors present on immune cells within the gut can be influenced by SSRIs, potentially altering immune responses and inflammation. These immune-mediated changes have the potential to affect the integrity of the intestinal barrier and its permeability.

In contrast to the harmful effects associated with chronic use of NSAIDs, PPIs, and SSRIs, vitamin E has been shown to confer protection toward the intestinal barrier. In a murine model of DSS-induced colitis, dietary supplementation with vitamin E prevented the depletion of the tight junction protein occludin, indicating enhanced barrier integrity.^168^ Additionally, owing to its recognized anti-inflammatory and antioxidant properties, vitamin E was hypothesized to mitigate the effects induced by TNF-α and IFN-γ treatment. In experiments using Caco-2 cell monolayers, α- and γ-tocopherol (natural derivatives of vitamin E) were found to preserve TEER levels and restore ZO-1 protein expression following cytokine exposure.^168^ While the precise mechanism underlying vitamin E’s protective action is not fully elucidated, studies indicating reduced expression of TLR-4 and NF-κB with vitamin E supplementation suggest its potential involvement in the established signaling pathways discussed previously in this review.^168,169^

Other natural compounds derived from sources beyond our microbiome, such as certain plant species, also play a role in modulating gut permeability. S*chisandra chinensis*, commonly known as the five-flavor fruit plant, has a rich history in herbal medicine, particularly for treating respiratory conditions.^170^ One of its derivatives, Schisandrin C, has demonstrated the ability to decrease FITC-dextran staining and enhance electrical resistance across Caco-2 cell layers exposed to IL-1β.^170^ Through its anti-inflammatory effects, Schisandrin C reduces the phosphorylation and nuclear translocation of NF-κB, while also reducing the expression of MLCK and p-MLC. These collective actions culminate in elevated levels of ZO-1 and occludin expression.^170,171^ Moreover, in an *in vivo* model using *C. elegans* infected with barrier-damaging bacteria, Schisandrin C demonstrated a reduction in FITC-dextran staining.^170^

Another noteworthy plant-derived compound is resveratrol, a natural polyphenol abundant in grapes, seeds, and berries.^172^ Renowned for its reported anti-cancer, anti-inflammatory, antioxidant, and neuroprotective effects, resveratrol modulates various signaling pathways involved in inflammation and tight junction protein expression.^172^ Despite some conflicting findings, resveratrol treatment in LPS-aggravated Caco-2 cells generally reduces inflammation by limiting NF-κB and TLR4 signaling.^173,174^ Additionally, resveratrol directly enhances tight junction protein expression, thereby reducing intestinal permeability. Treatment with resveratrol in cells with LPS-induced inflammation led to increased expression of ZO-1, occludin, and claudin-1.^175^ This increased expression is attributed to decreased inflammatory cytokine expression and attenuation of Notch-1 signaling, known inducers of barrier damage and modulators of tight junction proteins.^175^

Similarly, Aloe vera L. plant pulp, known for its antioxidative and anti-inflammatory effects, contains various polysaccharides and phytochemicals, with polysaccharides believed to be the primary therapeutic agent.^176,177^ Recent research demonstrates that processed gel from Aloe vera pulp replicates the beneficial effects of other naturally-derived polysaccharides on tight junction formation.^176^ Administration of Aloe vera gel reduced leakage *in vivo* and increased TEER and translation of tight junction proteins (ZO-1, occludin, claudin-1) *in vitro*.^176^ It is suggested that Aloe vera gel regulates tight junction protein expression by enhancing phosphorylation of ERK1/2 and activating MAPK/ERK signaling, another pathway influencing tight junction assembly.^176^

## 7. Concluding remarks

The integrity of the intestinal barrier, essential for maintaining gastrointestinal health, is intricately regulated by both intrinsic and extrinsic factors. Intrinsic factors, such as the composition of junctional complex proteins and intracellular signaling pathways acting on these proteins, play a fundamental role in maintaining the structural and functional integrity of the intestinal barrier. These factors ensure precise regulation of paracellular permeability, thereby preserving the selective permeability essential for nutrient absorption and defense against pathogens. Extrinsic factors, including diet, microbial communities, and environmental chemicals, significantly influence the function of intestinal junctional complexes. For instance, the gut microbiome profoundly impacts the modulation of junctional proteins, with beneficial microbes promoting barrier integrity and pathogenic microbes contributing to barrier dysfunction. Environmental factors, such as dietary components and xenobiotics, further interact with junctional complexes, either strengthening or compromising barrier function.

Disruptions in the intestinal barrier are implicated in various gastrointestinal and extraintestinal disorders, making it crucial to understand the complex interplay between these intrinsic and extrinsic modulators to elucidate the pathophysiology of these disorders. While the composition of intestinal junctional complexes is relatively well-studied, many open questions remain. For example:

- What biomarkers can be identified to reliably assess the integrity and function of intestinal junctional complexes in clinical settings?
- How can noninvasive techniques be developed or improved to monitor intestinal permeability and junctional complex function?
- What are the precise molecular mechanisms by which intrinsic factors, such as genetic mutations, epigenetic modifications, and intracellular signaling pathways, regulate the expression and function of junctional proteins?
- How do post-translational modifications (e.g., phosphorylation, ubiquitination) of junctional proteins influence their stability and function?
- How do specific microbial species and their metabolites influence the composition and function of intestinal junctional complexes?
- What are the mechanisms through which pathogenic microbes disrupt junctional complexes, and how can these pathways be targeted to prevent or treat barrier dysfunction?
- How does chronic exposure to environmental toxins and pollutants contribute to long-term changes in barrier function?
- How do intrinsic factors (e.g., genetic predispositions) modulate the response of the intestinal barrier to extrinsic factors (e.g., diet, microbiota, environmental chemicals)?
- What are the disease-specific alterations in junctional complexes that occur in conditions such as IBD, IBS, and other gastrointestinal disorders?
- How do systemic diseases, such as metabolic syndrome and autoimmune diseases, affect the regulation and function of intestinal junctional complexes?
- How can dietary interventions or probiotics/prebiotics be optimized to support and enhance the function of junctional complexes?

Addressing these critical questions will advance our understanding of the regulation and function of intestinal junctional complexes and their roles in health and disease. This knowledge will also inform the development of novel therapeutic strategies to maintain or restore intestinal barrier integrity.
